# The Effect of Park and Urban Environments on Coronary Artery Disease Patients: A Randomized Trial

**DOI:** 10.1155/2015/403012

**Published:** 2015-06-16

**Authors:** Regina Grazuleviciene, Jone Vencloviene, Raimondas Kubilius, Vytautas Grizas, Audrius Dedele, Tomas Grazulevicius, Indre Ceponiene, Egle Tamuleviciute-Prasciene, Mark J. Nieuwenhuijsen, Marc Jones, Christopher Gidlow

**Affiliations:** ^1^Department of Environmental Science, Vytautas Magnus University, K. Donelaicio 58, LT-44248 Kaunas, Lithuania; ^2^Department of Cardiology, Lithuanian University of Health Sciences, Eiveniu 2, LT-50167 Kaunas, Lithuania; ^3^Institute of Cardiology, Lithuanian University of Health Sciences, Eiveniu 2, LT-50167 Kaunas, Lithuania; ^4^Centre for Research in Environmental Epidemiology (CREAL), Doctor Aiguader 88, 08003 Barcelona, Spain; ^5^Centre for Sport, Health and Exercise Research, Staffordshire University, Brindley Building, Leek Road, Stoke-on-Trent ST4 2DF, UK

## Abstract

*Aim*. To test the hypothesis that walking in a park has a greater positive effect on coronary artery disease (CAD) patients' hemodynamic parameters than walking in an urban environment. *Methods*. Twenty stable CAD patients were randomized into two groups: 30-minute walk on 7 consecutive days in either a city park or busy urban street. Wilcoxon signed-rank test was employed to study short-term (30 min) and cumulative changes (following 7 consecutive days of exposure) in resting hemodynamic parameters in different environments. *Results*. There were no statistically significant differences in the baseline and peak exercise systolic blood pressure (SBP), diastolic blood pressure (DBP), heart rate (HR), exercise duration, or HR recovery in urban versus park exposure groups. Seven days of walking slightly improved all hemodynamic parameters in both groups. Compared to baseline, the city park group exhibited statistically significantly greater reductions in HR and DBP and increases in exercise duration and HR recovery. The SBP and DBP changes in the urban exposed group were lower than in the park exposed group. *Conclusions*. Walking in a park had a greater positive effect on CAD patients' cardiac function than walking in an urban environment, suggesting that rehabilitation through walking in green environments after coronary events should be encouraged.

## 1. Introduction

There is some evidence that green environments are associated with better self-reported health [1], lower blood pressure [2], lower psychophysiological stress [3, 4], and lower mortality risks [5]. However, the benefits of physical activity in green environments of CAD patients in terms of functional capacity are uncertain. Rehabilitation after coronary events, such as myocardial infarction, requires a specific approach to increase physical activity taking into account low cardiorespiratory fitness, impaired coronary flow reserve, and cardiac autonomic nervous system response [6–8]. The appropriate level of physical strain on the heart may improve these unfavourable changes. Long-term exercise training in patients with CAD is associated with a relative enhancement of vagal tone, improved HR recovery after exercise, and improved prognosis [9–13]. The effects of physical training in patients after acute MI on hemodynamic parameters may occur through improved autonomic nervous system function: HR recovery, resting HR, and SBP [8, 14–17]. Cardiac rehabilitation programmes include low- and moderate-intensity exercise such as walking. Regular walking has been shown to reduce anxiety and tension, improve cholesterol profile, and control blood pressure [18] and can help to lower SBP and DBP in hypertensive patients [19]. However, some authors have found that comprehensive rehabilitation after MI has no significant effect on risk factors, health-related quality of life, or physical activity [20]. The discrepancies between the studies' results may be a result of differences in study design and the environment where physical activity is conducted.

The underlying mechanisms for health benefits of green spaces are not fully understood. Recent studies have reported that green space, such as city parks, can reduce noise and air pollution [21, 22], enhance mood and related psychological outcomes [23], positively influence self-reported health [24–26], lower cumulative risk of cardiometabolic diseases [27], and lower metabolic syndrome scores [28].

There is some evidence that walking in a natural environment compared to an urban environment has benefits in terms of psychological and physical restoration in young subjects [2, 29] and also in hypertensive elderly patients [30]. Therefore we hypothesize that CAD patients walking in park will experience greater improvements in hemodynamic parameters than those walking in urban environment. Targeting patients with established CAD will have direct clinical applications for the use of different types of natural environment in cardiac rehabilitation. This study was conducted as part of EC FP7 PHENOTYPE project (Positive Health Effects of the Natural Outdoor Environment in Typical Populations in Different Regions in Europe) [31, 32]. This randomized study is the first to investigate whether the effect of walking for 30 min per day for seven days in a city park has greater positive impact on the CAD patients' hemodynamic parameters than walking in an urban environment.

## 2. Methods

### 2.1. Design of the Experiment

**Figure 1:**
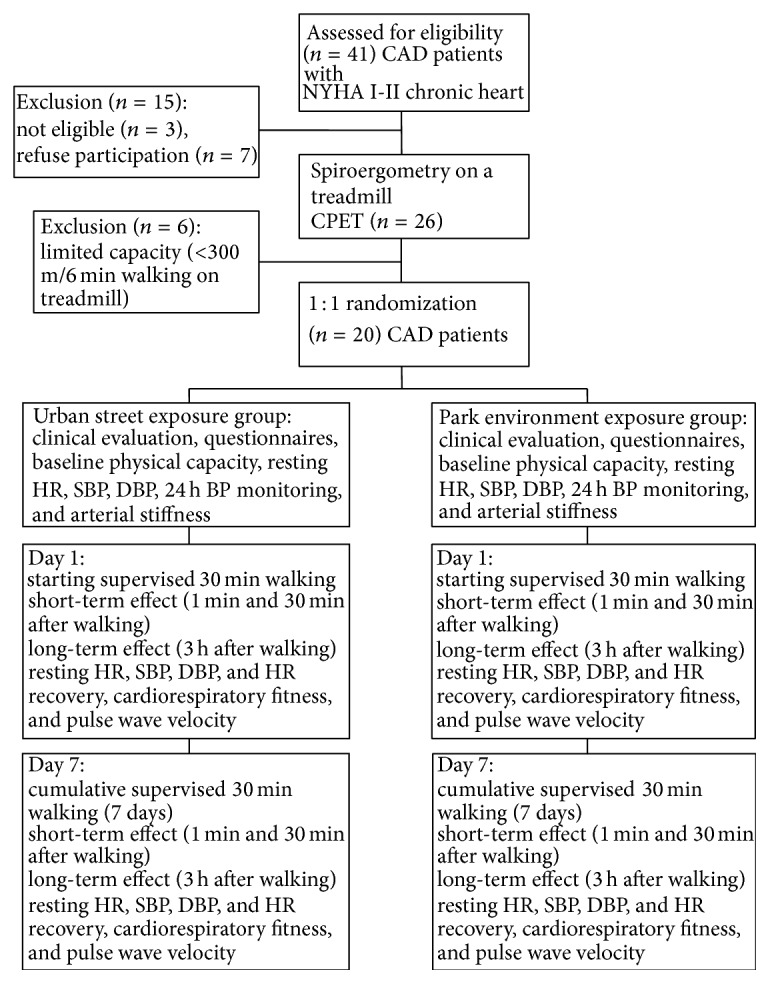
Flowchart, illustrating randomization and investigations scheme of urban street and park environment exposure groups.

The study was conducted in Kaunas, Lithuania. Twenty male and female Kaunas city residents (62.3 ± 12.6 years of age) with CAD (functional class by the New York Heart Association (NYHA) I-II chronic heart failure) participated in the study. The patients were treated at the Cardiologic Clinic of the Hospital of Lithuanian University of Health Sciences because of MI or unstable angina pectoris and were consecutively selected from the patients register. The mean duration since the last period of CAD hospitalization and cardiac rehabilitation was 1.03 ± 0.5 years. Inclusion criteria were as follows: 45–75 years of age, men or women, who survived MI or unstable angina pectoris, and signed informed consent to take part in the study. Exclusion criteria were as follows: unstable angina pectoris, cardiomyopathy, idiopathic or organic valvular disease, hypertension with SBP > 160/110 mm Hg, diabetes mellitus type 2, electrocardiostimulation, neurological diseases, and limited capacity (less than 300 m achieved after 6 min walking on treadmill) (Figure 1).

The study was performed under the regulations of the Lithuanian Bioethics Committee and in accordance with the Declaration of Helsinki.

### 2.2. Study Protocol

Patients were randomly assigned to either green or urban exposure groups. The urban exposure (*n* = 10) was a busy street behind the Cardiology Clinic (10,000 cars/d). The green exposure (*n* = 10) was a pine park located within a 5 min walk of the Cardiology Clinic, accessed through clinic park (in total 30 min green exposure). Patients' normal medication regimens were not changed during the study. We used standardised protocols for environmental exposure and measurement of physiological responses. Both groups were similar, clinically and in terms of their residential environmental characteristics. Physical activity, eating, and drinking were controlled during the study periods. Data collection took place at the clinic between 12:00 and 15:00, May–September 2013. To minimise the social interaction effects during the environmental exposure, the same trained researcher supervised all subjects, their walking intensity, and their social interaction during the 30 min walk. Exercise capacity testing using a spiroergometer on a treadmill and with ECG monitoring was performed at baseline and day 7. The test provides an accurate assessment of maximal and functional aerobic capacity. Walking intensity was estimated to be 10% lower of the capacity determined during spiroergometry. Patients walked for 30 min each day in their allocated environment, for 7 consecutive days. We studied the short-term (1 min and 30 min after walk) and cumulative 7-day effects of walking alone in the urban or park environment and 7-day changes in specific exercise capacity parameters. Changes in hemodynamic parameters at rest and at peak exercise (HR, SBP, and DBP, cardiorespiratory fitness, and HR recovery) were assessed. Before and after 7 days of walking in different environments, we compared changes in hemodynamic parameters between those walking in the urban and the park environments including resting data before walking, 1 min after walking, 30 min after walking, and 3 hours after walking.

### 2.3. Measurements

On the day prior to the experiments, subjects before signing the Informed Consent Form were informed of the aims and procedures and then completed the standard PHENOTYPE questionnaires and took part in the 1st laboratory test to estimate baseline physical capacity. The standard questionnaires included questions regarding the respondent's personal characteristics, wellbeing and health, health behaviour, CAD anamnesis, residence history, and neighbourhood. We used the CS-200 Schiller spiroergometer on a treadmill following the Naughton protocol, after evaluation of indication and contraindication for the exercise test [33]. We evaluated cardiac autonomic nervous system effects on hemodynamic parameters by measuring resting HR, SBP, DBP, and HR recovery following exercise [11, 16]. Resting cardiovascular parameters were measured in a seated position at least 15 min before the start of the spiroergometric testing. At baseline, the exercise intensity was determined according to the baseline HR at the individual level of the ventilatory level threshold, assessed by spiroergometry. The treadmill exercise test began at 3 km/h with a 10% incline. This increased every 3 min by 1.8 km/h and 2% incline. HR recovery was estimated by difference between HR at peak exercise and HR 1 min after completion of exercise. The exercise was terminated when the patients reached 75% of their maximal HR or displayed limiting symptoms (chest pain or pressure, dizziness, dyspnoea, weakness) or ST depression on the ECG of more than 2 mm. This was followed by a 10 min recovery and assessment of exercise capacity and cardiovascular parameters. To estimate physical capacity in W, we measured workload which refers to the work done with a given load and total energy output. We assessed changes in physical capacity by the cardiopulmonary exercise test before and after 7 days of different environment exposures. On day 1 and day 7, arterial distensibility was estimated by pulse wave velocity (PWV) using SphygmoCor. Subjects also completed 24-hour ambulatory BP monitoring (ABPM) using the Microlife WatchBP 03, which took measures at 15 min intervals during the day (09:00–21:00) and at 30 min intervals overnight. Peak SBP and DBP were recorded as the highest values achieved when walking in the different environments.

### 2.4. Statistical Analysis

We used the exact Fisher tests to compare the personal characteristics of the urban and park exposed patients. Quantitative variables are reported as means and standard error. Quantitative clinical and environmental variables in both groups were compared using the Mann-Whitney* U* test and the chi-square test. We used nonparametric tests because the data were not normally distributed. The Wilcoxon signed-rank test was used to compare measurements before and after each walk and between day 1 and day 7. The level of statistical significance was *P* < 0.05. All statistical analyses were performed using SPSS version 18.0 (SPSS Inc. Released 2009; PASW Statistics for Windows, Version 18.0; Chicago: SPSS Inc).

## 3. Results

There were no statistically significant differences in the demographic or clinical characteristics between the two exposure groups (Table 1).

The mean age, body mass index (BMI), duration of CAD anamnesis, and time since last CAD hospitalisation were all similar for patients in the urban and park groups. Residential environmental characteristics were also similar: the mean residential NO_2_ concentration of patients exposed to urban environment was 18.5 ± 5.4 *μ*g/m^3^ and that exposed to park environment was 20.1 ± 5.3 *μ*g/m^3^ (*P* = 0.37), while the residential proximity to the nearest city park was 321.7 ± 251 m and 490 ± 356 m, respectively (*P* = 0.114). There were significant differences in the characteristics of the urban versus park environments, with higher levels of air pollution (NO_2_ concentration 3.84 *μ*g/m^3^ higher, PM2.5 6.41 *μ*g/m^3^ higher) and noise (19.03 dBA higher) compared with the park environment.

The two groups did not differ significantly in terms of mean SBP, DBP, and HR before exposure (Table 2).

Baseline exercise capacity testing, which provides an accurate assessment of maximal and functional aerobic capacity, showed that there was no significant difference between the urban and park exposure groups for exercise duration (where longer duration indicates greater capacity) (4.97 ± 1.43 and 5.66 ± 0.80 min, *P* = 0.205, resp.), work load, or postexercise HR recovery. Pulse wave velocity was also similar. After 7 days of walking there were no statistically significant changes between the urban and park groups in terms of hemodynamic parameters. However, work load was slightly higher (169.3 W and 215.4 W, *P* = 0.076), and SBP and DBP were slightly lower in park group. Both groups demonstrated slight decreases in HR and increases in exercise capacity test duration and HR recovery as a consequence of regular walking. There were no statistically significant changes between the groups in pulse wave velocity (*P* = 0.475).

Analysis of short-term (1 min and 30 min after exercise) changes in hemodynamic parameters on days 1 and 7 revealed statistically significant differences in hemodynamic indices at 1 min after walking compared with baseline (Table 3). On day 1, 1 min after walking, patients in both groups had higher SBP and HR than at baseline, and higher DBP was evident in the urban group. After 30 min rest, SBP, DBP, and HR decreased to baseline levels in both groups. On day 7, 1 min after walking, increases observed in HR (from baseline levels) were significantly lower for the park exposure than the urban exposure group. After a week of exposure in both groups, increases in SBP and HR measured 1 min after walking were again evident but decreases to baseline levels in the hemodynamic parameters at 30 min postexposure were found only in those exposed to park environment; that is, those walking in green environments showed faster favourable hemodynamic changes compared with the urban group. When we tested for significance of these apparent differences, on day 1, 1 min after walking only DBP differed (due to the slower reduction in DBP from a higher postexposure level in the urban group). The difference in DBP on day 7, 30 min after walking, was statistically significant between the urban and park groups (+4.0 and −2.4 mm Hg, resp., *P* = 0.045).

The difference in resting hemodynamic parameters measured at baseline of days 1 and 7 is presented in Table 4. After seven days of exposure, we found a slight decrease in resting DBP and HR before the exercise test and a decrease in resting HR three hours after the test (mean value derived from ambulatory monitoring) in patients exposed to urban environment. However, there was evidence of a positive training effect on hemodynamic parameters in patients exposed to park environment; on day 7, three hours after exercise we found a stable and statistically significant decrease in SBP (6.50 mm Hg) and DBP (6.29 mm Hg) compared with pretraining data (*P* = 0.049 and *P* = 0.014, resp.). Significant increases in exercise duration (increase of 1.1 min, *P* = 0.004) and HR recovery (5.89 beats/min, *P* = 0.037) were also observed in the park group, while in urban environment exposed patients, changes in these parameters were not statistically significant.

## 4. Discussion

The present study aimed to use objective measures to assess the physiological effects of controlled walking in urban and park environments in CAD patients. Data showed that regular 30 min walks of moderate intensity in a park environment performed on 7 consecutive days led to greater favorable changes in resting SBP and DBP, improvements in exercise tolerance, and increases in exercise duration, compared with equivalent walks in an urban environment. Walking in the park also increased patients' HR recovery after everyday physical exercise. Because HR recovery (fall in HR 1 min after exercise) is treated as an indicator of autonomic function [8, 16, 17], the increase in HR recovery could be the result of improved autonomic nervous function regulation induced by physical training in green environment. The results presented offer some support for our hypothesis that walking in the park environment has better restorative effect on impaired hemodynamic in CAD patients compared with walking in a busy urban street.

To our knowledge, no previous studies have compared the effects of controlled walking in urban and park environments on hemodynamic parameters in CAD patients. Our results are consistent with evidence from healthy young adults. The comparison of physiological effects of 15 min of walking in forest and urban environment in 12 Japanese students revealed significantly lower SBP, DBP, and HR and higher HR variability in subjects exposed to a forest environment showing suppressed sympathetic nervous activity and enhanced parasympathetic nervous activity in the forest area [34]. The greater positive effect on young adults BP during and after 30 min walking was found among those exposed to a green environment versus urban environment [2]; however, the effect soon disappeared after walking. The study of forest walking in young Japanese males showed cardiovascular relaxation, decreased SBP, lower HR, and reduced negative psychological symptoms in the forest environment exposed young males. These results suggested that physical activities in park environment can promote cardiovascular relaxation [35].

In our study improvements observed in exercise tolerance and increased HR recovery after 7 days of 30 min walks in a park environment may be explained by the positive influence of forest-related activities on cardiovascular relaxation and recovery of homeostasis in CAD patients. This mechanism may be partially confirmed by the findings of young Japanese adult males, indicating that walking in the forest environment can facilitate homeostasis [35]. Physiological studies support that green environment effects can manifest on homeostasis through positive effects on the central and autonomic nervous systems and endocrine systems [34].

Our findings are in accordance with the results of epidemiological studies, which show positive relationships between the physical activity in natural environment and cardiovascular health. A Kaunas cohort study that investigated associations between the accessibility and use of urban city parks and cardiovascular health showed that the prevalence of cardiovascular risk factors was statistically significantly lower among park users than among nonusers. Men living further away from parks and rarely using them had a higher risk of nonfatal and fatal CVD combined, compared with those living nearby; that is, regular use of green space in a city setting was linked to reduced risk of heart disease [36]. An observational study in Perth, Western Australia [37], showed that higher greenness level within a neighbourhood was associated with lower heart disease or stroke risk, and a randomized controlled trial [38] indicates that even short exercise-based rehabilitation may improve long-term outcomes.

In our study, differences in measured characteristics of the two environments may also partially explain our findings. During walking in the urban street, NO_2_ was higher by 3.84 *μ*g/m^3^, PM2.5 by 6.41 *μ*g/m^3^, and noise level by 19.03 dBA (compared with the park environment). Such differences may have impact on psychophysiological stress, homeostasis, and hemodynamic parameters. Previously published data from Kaunas [39, 40] and studies elsewhere [41, 42] indicate that such an increase of urban NO_2_ pollution, noise level, and PM2.5 pollution may increase the risk of hypertension and that this, through increase in SBP and DBP, may promote atherosclerosis and CAD. Short-term increases in exposure to ambient PM2.5 are associated with acute increases in blood pressure in adults [43]. The particle pollution in CAD patients during physical activity may increase systemic arterial vascular narrowing, as manifested by increased peripheral blood pressure and HR [44, 45], and promote arterial vasoconstriction via altering cardiovascular autonomic nervous system balance [46–48]. These findings support our conclusions that physical activity in the park environment has a greater positive impact on cardiovascular health than physical activity in an urban street and that to increase the efficacy of exercise-based cardiac rehabilitation for urban residents, walking in green environments should be recommended.

The study results will have direct practical applications for the use of natural environments in cardiac rehabilitation. However, some limitations are recognised. First, the sample size was relatively small, albeit large enough to detect some significant effects. Second, we are unable to identify which specific characteristics of the natural and urban environments were responsible for the observed effects. During walking, patients were affected not only by the traffic emissions but also by the view of trees planted in front of the houses and that may have impact on the decrease of psychophysiological stress level and obtained results.

These limitations notwithstanding, this study appears to be the first to analyse the relationship between the controlled physical activity in different environments and CAD patient's hemodynamic parameters, further adding to the growing support for the therapeutic potential of natural environments. Natural environments should be considered for inclusion in physical rehabilitation after CAD, but further research with larger samples is required to draw generalized scientific conclusions on the impact of natural environmental quality on CAD patients.

## Figures and Tables

**Table 1:** Baseline characteristics of the urban street and park environment study groups (data shown as mean values ± standard deviation or numbers and percentages).

Baseline characteristics	Urban street mean ± SD	Park environment mean ± SD	*P* ^*∗*^
Men	6 (60%)	7 (70%)	0.500
Age, years	66.0 ± 12.5	58.5 ± 12.2	0.162
Body mass index, kg/m^2^	27.9 ± 1.8	27.9 ± 4.9	0.264
CAD anamnesis, years	9.3 ± 8.8	8.8 ± 11.7	0.353
Duration after the last CAD hospitalization, years	1.16 ± 0.6	0.90 ± 0.4	0.176
NO_2_ in living environment, *µ*g/m^3^	18.5 ± 5.4	20.1 ± 5.3	0.370
Residence proximity to park, m	321.7 ± 251	490 ± 356	0.114
NO_2_ during walking, *µ*g/m^3^	24.15 ± 1.69	20.31 ± 0.93	0.026
PM2.5 during walking, *µ*g/m^3^	24.64 ± 0.97	18.23 ± 0.85	0.001
Noise during walking, dBA	65.20 ± 1.31	46.17 ± 0.78	0.000

^*∗*^Exact one-tailed *P* value of Mann-Whitney *U* test.

**Table 2:** Comparison of baseline and the seventh day exposure hemodynamic data at rest and at peak exercise as mean (SE) in patients of urban or park environment exposure.

Measurements	Urban exposure Mean (SE)	Park exposure Mean (SE)	*P* ^*∗*^ value
First day baseline			
Systolic BP baseline, mm Hg	134.7 (6.8)	135.9 (5.5)	0.382
Diastolic BP baseline, mm Hg	80.3 (3.3)	81.4 (1.7)	0.398
Heart rate baseline, beats/min	77.7 (4.0)	71.3 (3.8)	0.125
Peak SBP, mm Hg	181.4 (6.5)	191.2 (4.2)	0.133
Peak DBP, mm Hg	94.1 (3.1)	94.3 (1.6)	0.232
Peak heart rate, beats/min	125.1 (6.7)	139.7 (4.5)	0.039
Exercise duration, min	4.97 (1.43)	5.66 (0.80)	0.205
Work load, W	159.5 (24.9)	184.8 (26.0)	0.144
Heart rate recovery, beats/min	20.6 (5.6)	23.4 (2.7)	0.122
Pulse wave velocity m/s	9.94 (0.8)	9.67 (1.0)	0.452
Seventh day baseline			
Systolic BP baseline, mm Hg	135.9 (5.7)	131.2 (6.0)	0.217
Diastolic BP baseline, mm Hg	80.2 (3.9)	77.2 (2.7)	0.324
Heart rate baseline, beats/min	76.1 (4.1)	70.0 (3.2)	0.163
Peak SBP, mm Hg	186.9 (7.4)	187.4 (5.5)	0.340
Peak DBP, mm Hg	94.1 (3.1)	90.0 (2.9)	0.209
Peak heart rate, beats/min	127.8 (6.8)	139.2 (5.5)	0.043
Exercise duration, min	5.23 (1.31)	6.69 (0.90)	0.158
Work load, W	169.3 (32.0)	215.4 (26.3)	0.076
Heart rate recovery, beats/min	27.4 (3.5)	31.0 (2.9)	0.152
Pulse wave velocity m/s	10.3 (0.9)	10.0 (1.0)	0.475

^*∗*^Exact one-tailed *P* value of Mann-Whitney *U* test.

**Table 3:** The difference in hemodynamic parameters between baseline and 1 min and baseline and 30 min after walking in urban or park environment on the first and the seventh day.

Measurements	Difference 1 min after walking		Difference 30 min after walking			
	Mean (SE)	*P* ^*∗*^ value	*P* ^*∗∗*^ value	Mean (SE)	*P* ^*∗*^ value	*P* ^*∗∗*^ value
Urban exposure						
SBP, mm Hg day 1	14.5 (3.3)	0.008		2.3 (4.4)	0.199	
DBP, mm Hg day 1	10.1 (2.5)	0.008		2.0 (2.5)	0.148	
HR, b/min day 1	23.1 (6.5)	0.004		−7.6 (5.6)	0.125	
SBP, mm Hg day 7	19.1 (5.5)	0.010		12.8 (6.8)	0.064	
DBP, mm Hg day 7	6.1 (4.3)	0.150		7.4 (3.9)	0.023	
HR, b/min day 7	28.3 (4.9)	0.002		10.9 (5.1)	0.037	
Green exposure						
SBP, mm Hg day 1	11.4 (5.7)	0.035	0.236	7.2 (6.7)	0.125	0.483
DBP, mm Hg day 1	1.7 (2.3)	0.227	0.018	3.6 (6.7)	0.086	0.264
HR, b/min day 1	15.1 (4.7)	0.010	0.223	3.6 (4.1)	0.275	0.152
SBP, mm Hg day 7	22.3 (5.2)	0.002	0.389	5.0 (5.2)	0.172	0.091
DBP, mm Hg day 7	4.0 (3.9)	0.238	0.356	−2.4 (3.2)	0.258	0.045
HR, b/min day 7	12.7 (4.0)	0.008	0.015	5.3 (4.2)	0.172	0.252

^*∗*^Exact one-tailed *P* value of Wilcoxon test between baseline and 1 min after exposure.

^**^Exact one-tailed *P* value of Mann-Whitney *U* test between exposure groups.

**Table 4:** The changes (mean (SE)) of hemodynamic parameters between the first and the seventh day exposure in urban and park environments.

Measurements at day 1 and day 7	Urban exposure changes in mean (SE)	*P* ^*∗*^ value	Park exposure changes in mean (SE)	*P* ^*∗*^ value
SBP, mm Hg before test	1.22 (3.9)	0.336	−4.70 (6.0)	0.456
DBP, mm Hg before test	−0.11 (2.3)	0.453	−4.20 (2.2)	0.031
HR, b/min before test	−1.56 (1.9)	0.348	−1.3 (3.3)	0.500
SBP, mm Hg 3 h after test	1.30 (2.8)	0.469	−6.5 (3.7)	0.049
DBP, mm Hg 3 h after test	1.93 (3.8)	0.422	−6.29 (2.4)	0.014
HR, b/min 3 h after test	−4.16 (3.5)	0.172	−1.79 (1.6)	0.188
Peak SBP, mm Hg	5.5 (3.2)	0.156	−3.8 (5.8)	0.262
Peak DBP, mm Hg	0 (2.3)	0.453	−4.3 (3.3)	0.234
Peak heart rate, b/min	2.63 (4.0)	0.223	0.33 (3.7)	0.422
Exercise duration, min	0.26 (0.3)	0.230	1.10 (0.28)	0.004
Work load, W	9.8 (9.8)	0.500	30.9 (13.0)	0.063
Heart rate recovery, b/min	6.75 (4.5)	0.121	5.89 (2.6)	0.037
Pulse wave velocity m/s	0.37 (0.9)	0.410	0.35 (0.8)	0.321

^*∗*^Exact one-tailed *P* value of Wilcoxon test.

## Abbreviations

CAD: Coronary artery disease
HR: Heart rate
SBP: Systolic blood pressure
DBP: Diastolic blood pressure
min: Minute.
